# Supplementary figures and images for: Acitretin mitigates uroporphyrin-induced bone defects in congenital erythropoietic porphyria models
Source: Sci Rep. 2021 May 5;11:9601. doi: 10.1038/s41598-021-88668-9 (PMC8100164; doi:10.1038/s41598-021-88668-9)

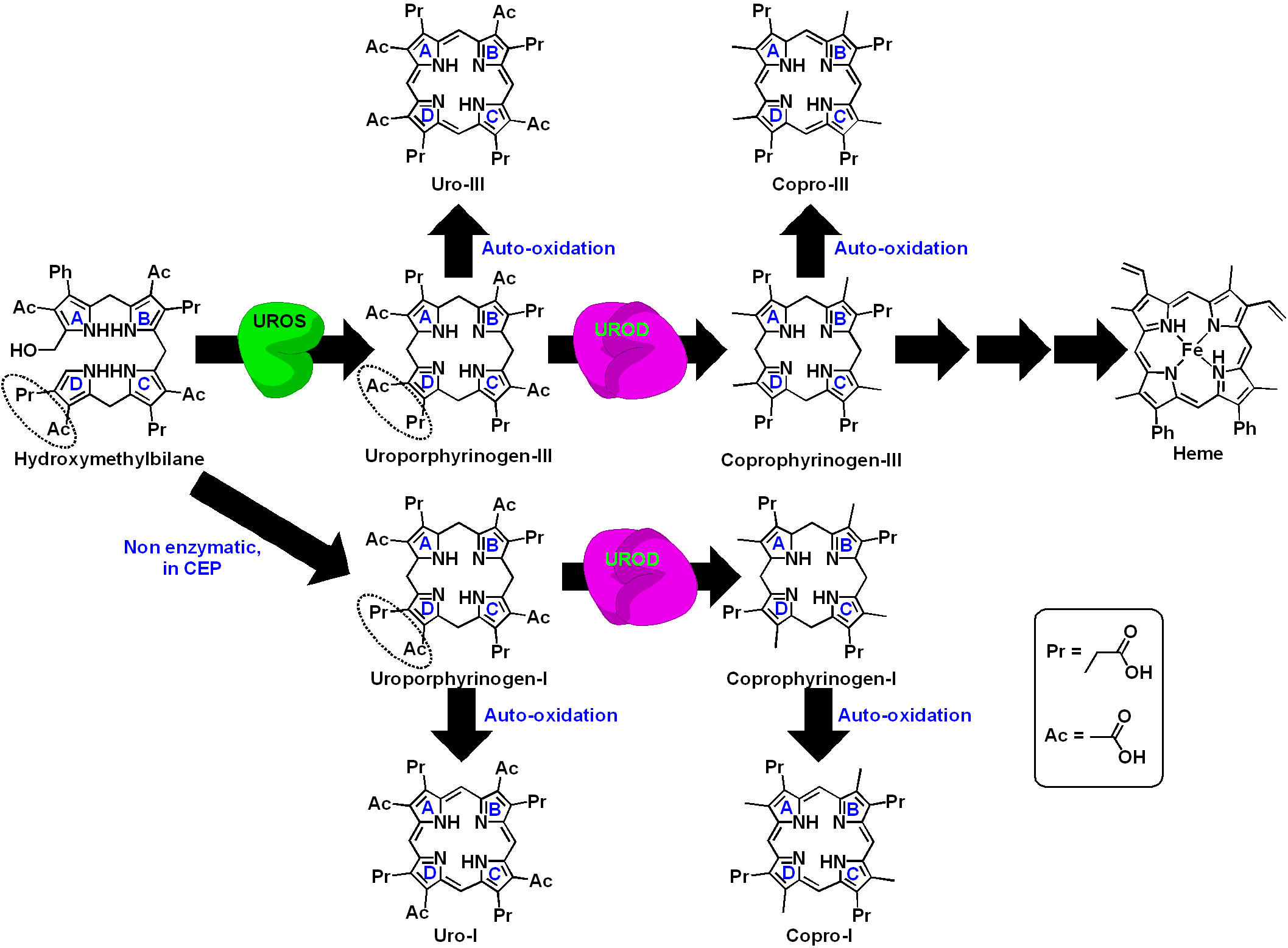

Supplement: Supplementary file 1 — Supplementary Information 1. [file 41598_2021_88668_MOESM1_ESM.tif]

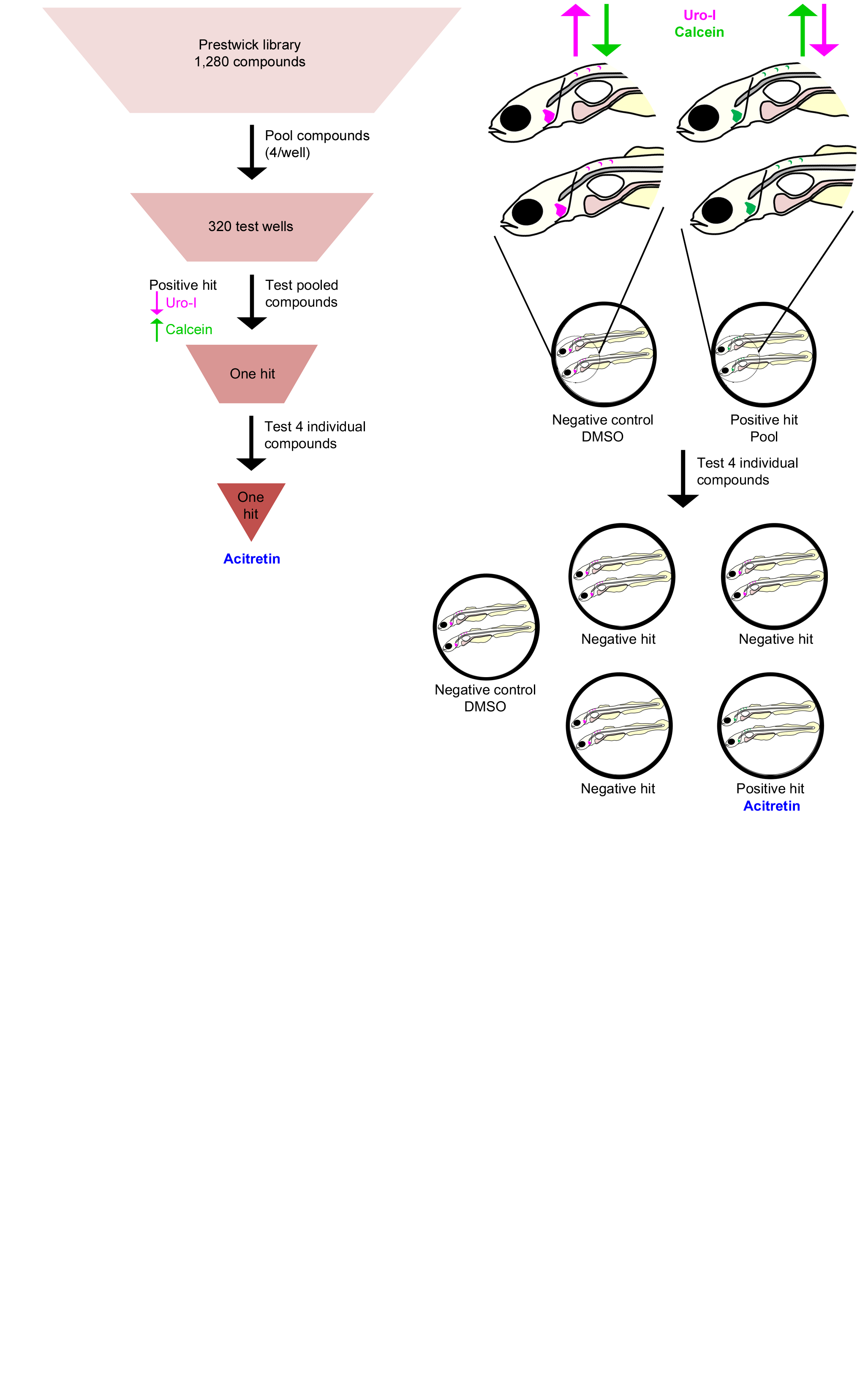

Supplement: Supplementary file 2 — Supplementary Information 2. [file 41598_2021_88668_MOESM2_ESM.tif]

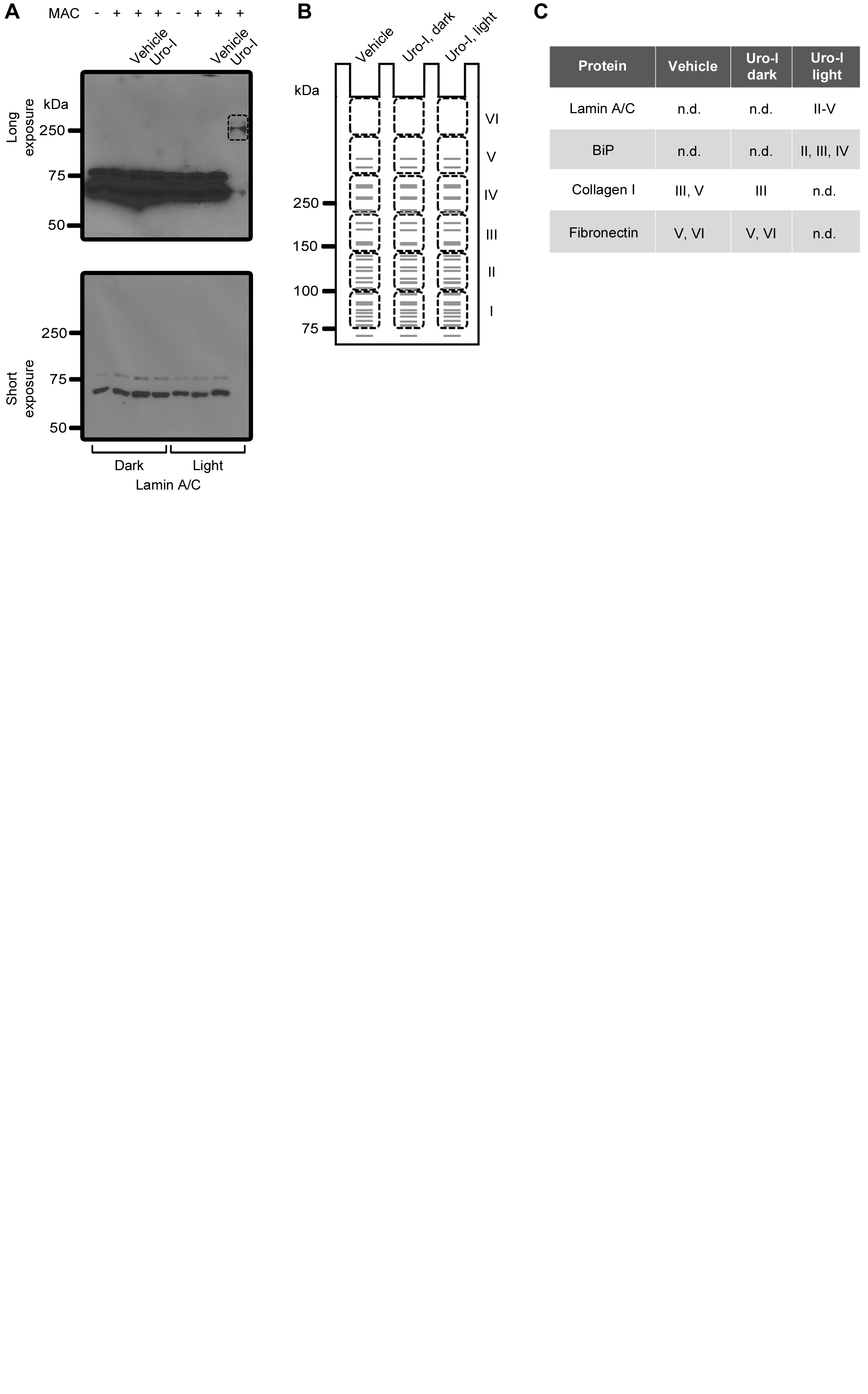

Supplement: Supplementary file 3 — Supplementary Information 3. [file 41598_2021_88668_MOESM3_ESM.tif]

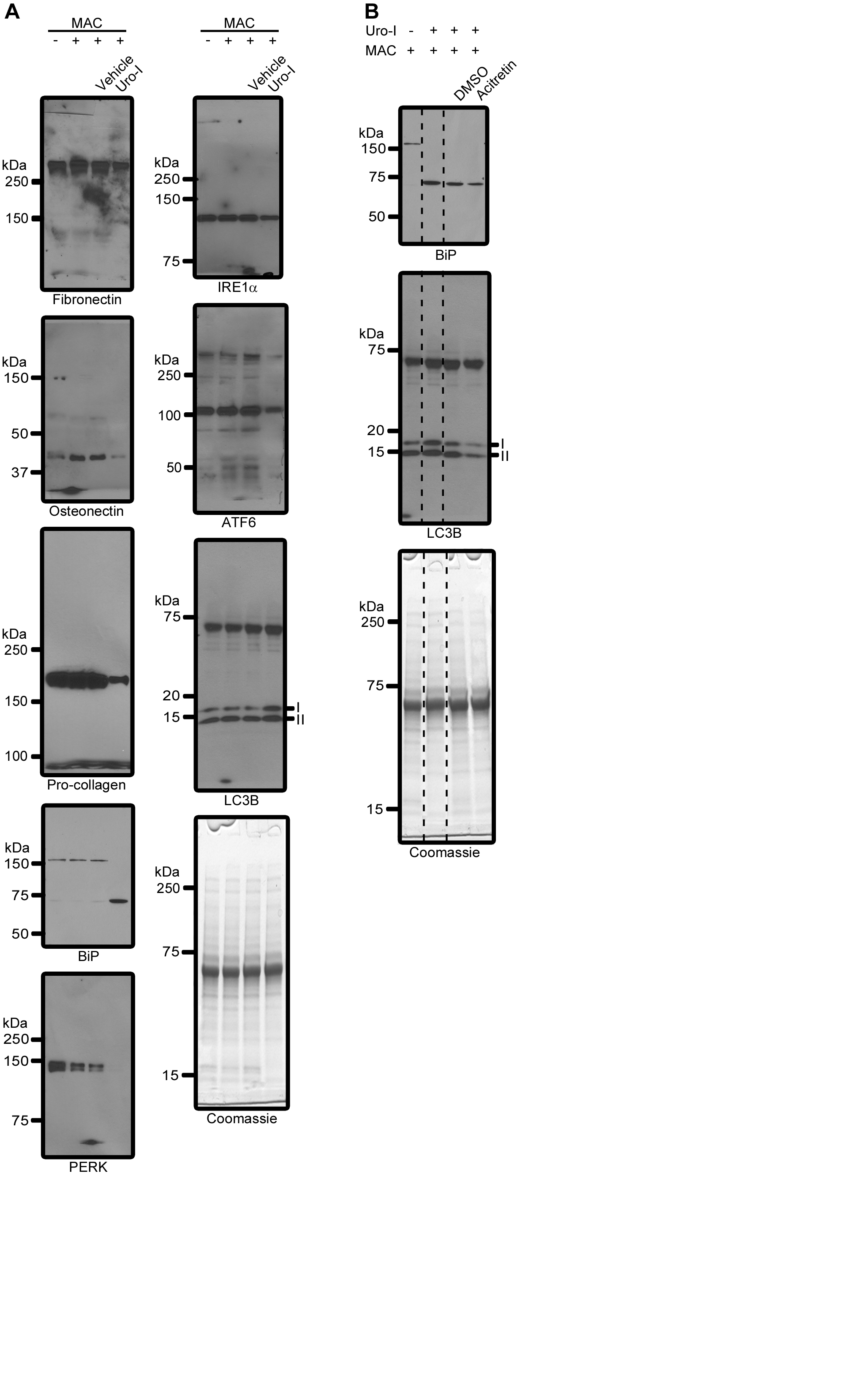

Supplement: Supplementary file 4 — Supplementary Information 4. [file 41598_2021_88668_MOESM4_ESM.tif]
