# Supplementary material for: Acitretin mitigates uroporphyrin-induced bone defects in congenital erythropoietic porphyria models
Source: Sci Rep. 2021 May 5;11:9601. doi: 10.1038/s41598-021-88668-9 (PMC8100164; doi:10.1038/s41598-021-88668-9)
Supplement: Supplementary file 5 — Supplementary Information 5. [file 41598_2021_88668_MOESM5_ESM.docx]

**Acitretin mitigates uroporphyrin-induced bone defects in congenital erythropoietic porphyria models**

Juliana Bragazzi Cunha^1#*^, Jared S Elenbaas^2#^, Dhiman Maitra^1#^, Ning Kuo^1^, Rodrigo Azuero-Dajud^1^, Allison C Ferguson^3^, Megan S Griffin^3^, Stephen I Lentz^4^, Jordan A Shavit^3^, M Bishr Omary^1,5*^

^1^Center for Advanced Biotechnology and Medicine, Rutgers University, Piscataway, 08854

^2^Medical Scientist Training Program, Washington University, Saint Louis, 63110

^3^Department of Pediatrics, Division of Pediatric Hematology/Oncology, University of Michigan, Ann Arbor, 48109

^4^Department of Internal Medicine, Division of Metabolism, Endocrinology and Diabetes, University of Michigan, Ann Arbor, 48109

^5^Department of Molecular and Integrative Physiology, University of Michigan Medical School, Ann Arbor, 48109

^#^Equal contribution

***Corresponding author:** B Omary ([bo163@cabm.rutgers.edu](mailto:bo163@cabm.rutgers.edu)) and J Bragazzi Cunha ([bragazzi@umich.edu](mailto:bragazzi@umich.edu))

**Running title:** Acitretin protects from CEP damage


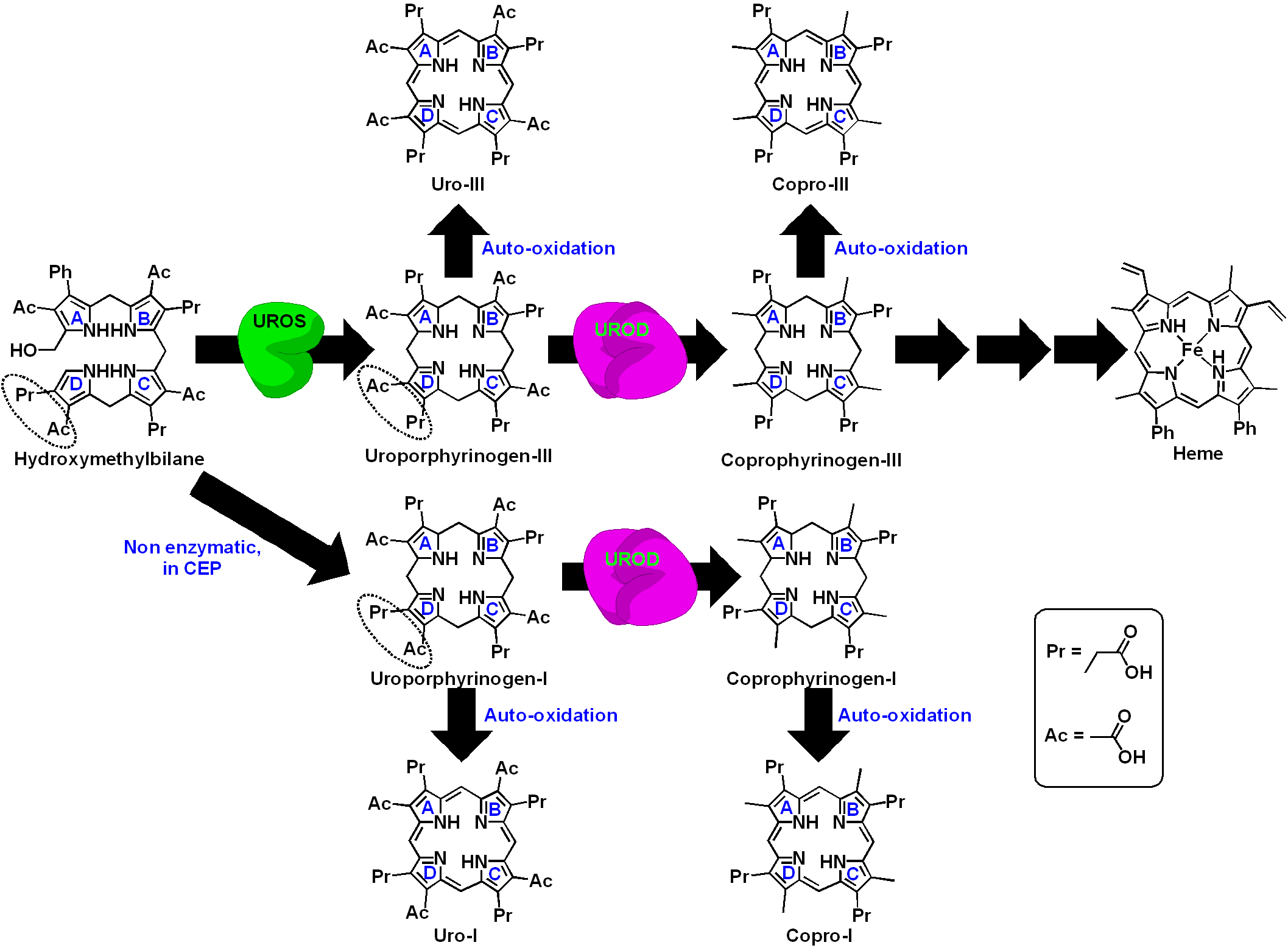


Figure S1. Uroporphyrinogen III synthase (UROS) inhibition accumulates uro-I and copro-I in CEP. UROS, a cytosolic enzyme, catalyzes the conversion of the linear tetrapyrrole, hydromethylbilane (HMB) to the first cyclic tetrapyrrole of the pathway, uroporphyrinogen-III^1,2^. UROS ‘flips’ the position of the acetate and propionate in the ‘D’ pyrrole ring and subsequently causes ring closure to form uroporphyrinogen-III (dotted oval)^1,3^. Uroporphyrinogen-III is decarboxylated by uroporphyrinogen decarboxylase (UROD) to form coproporphyrinogen-III, which through a multi-step mechanism that involves the formation of protoporphyrin-IX, generates heme. In absence of UROS activity, there is spontaneous ring closure of HMB to form uroporphyrinogen-I, a positional isomer of uroporphyrinogen-III, where the acetate/propionate inversion in ring ‘D’ does not occur. Uroporphyrinogen-I is decarboxylated by UROD to coproporphyrinogen-I, but after this step the pathway gets blocked since coproporphyrinogen-I cannot be metabolized by coproporphyrinogen oxidase. Porphyrinogens are relatively unstable compounds, and are auto-oxidized from their colorless, non-fluorescent porphyrinogen forms to colored, fluorescent porphyrins^4^. Thus UROS blockade leads to accumulation of uroporphyrin-I (uro-I) and coproporphyrin-I (copro-I).


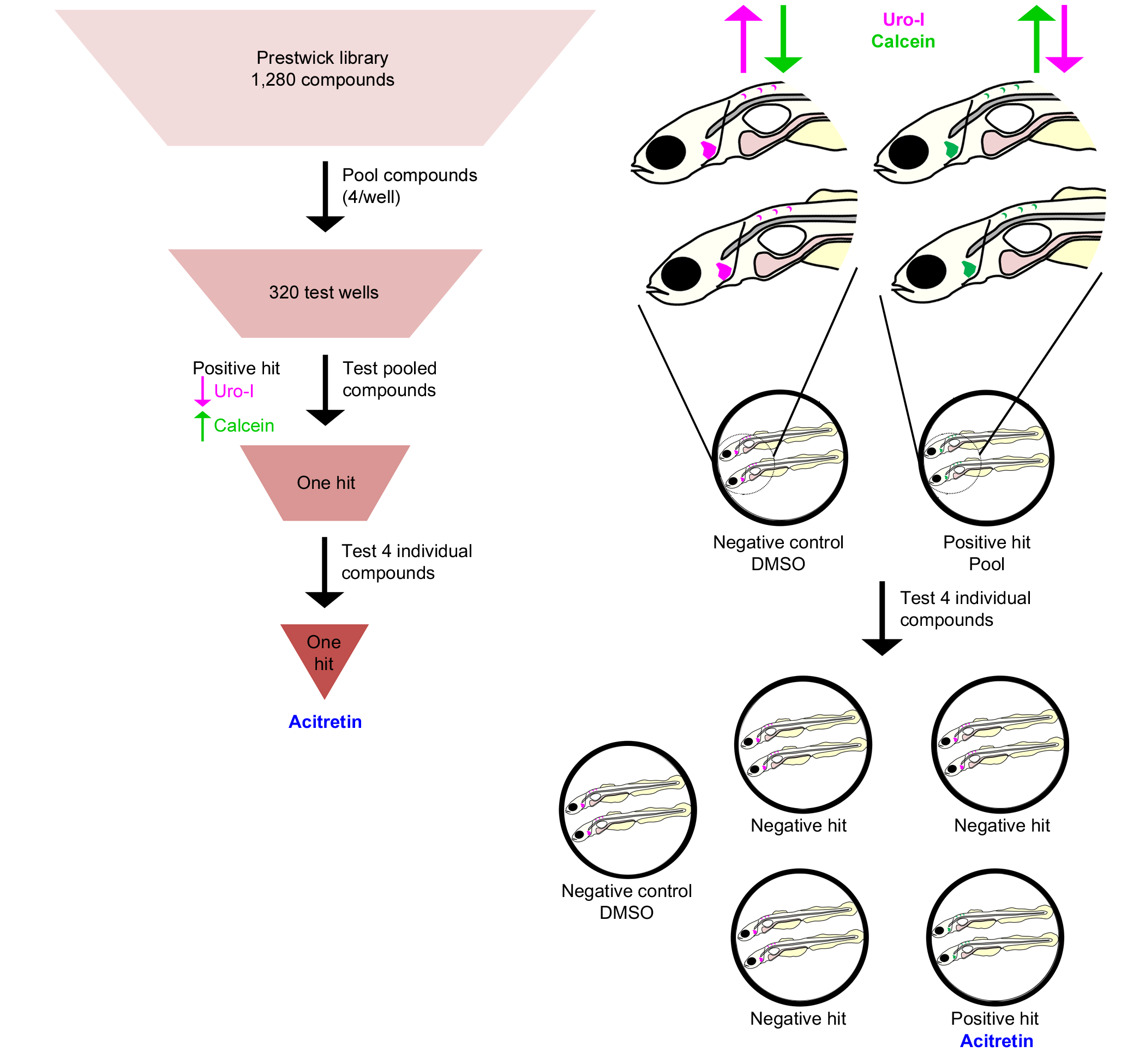


Figure S2. High throughput drug screening for CEP. High throughput drug screening protocol to identify potential drug treatments for CEP was conducted by testing 1,280 small molecules from the commercially available Prestwick library. Initial screening was performed by pooling four drugs per well, with two zebrafish larvae in each well. 6dpf zebrafish larvae were injected with uro-I and calcein simultaneously. 24h later, they were imaged by epiflourescence microscopy using the automated ImageXpress system. Visual analysis was conducted and identification of wells containing larvae with reduced uro-I and increased calcein signal (magenta and green arrows, respectively) in bones compared to DMSO-treated larvae were selected for individual testing of each drug. Of the 320 pools tested, one was identified as potential hit. Once the four drugs were tested individually, acitretin was identified for decreasing uro-I accumulation in bones.


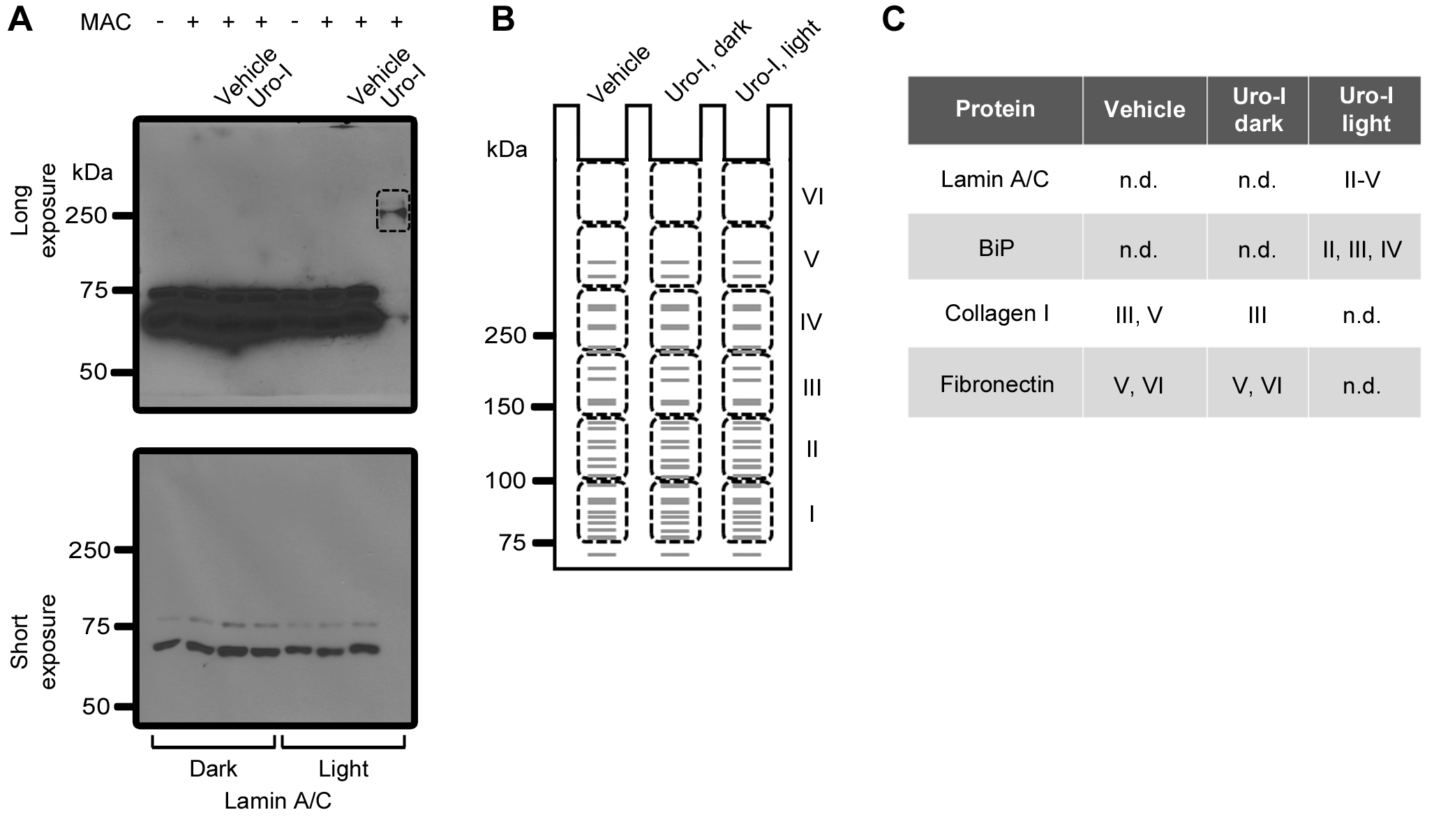


Figure S3. Uro-I causes aggregation of bone matrix proteins in a light-independent manner. (A) Saos-2 cells were treated for three days with uro-I or vehicle in the presence of mineralization activation cocktail (MAC). Cells grown in medium without MAC (no mineralization stimuli) and in MAC alone were used as controls for MAC efficiency. Experiments were performed in a dark room and cells were shielded from light throughout the whole experiment. In order to verify whether protein aggregation took place while cells were alive and represented a biologically relevant finding, or if aggregation was an artifact of light exposure during processing of samples, an aliquot of lysate from uro-I treated cells was exposed to light prior to addition of reducing SDS-PAGE sample buffer, which we have shown previously that prevents light-induced protein aggregation by porphyrins in cell lysate. Uro-I treatment did not cause lamin A/C to aggregate, with monomer being comparable between vehicle- and uro-I treated cells (3rd and 4th lanes, short exposure). However, upon light exposure of the uro-I treated cells lysate, loss of monomer and high molecular aggregates were observed (7th and 8th lanes, long exposure). These findings confirm that accidental light exposure of samples did not happen, and any protein aggregation observed was a true biological event, not an artifact of cell processing. (B) We conducted a proteomics experiment of cell lysates treated with uro-I and vehicle in the dark to further confirm our findings that bone matrix proteins aggregated upon uro-I treatment. Six 1cm regions of a coomassie stained gel (I-IV, cartoon) spanning from the bottom of the well to slightly above the 75kDa marker were cut and submitted to mass spectrometry analysis. (C) Our results confirmed lamin A/C aggregated only in the light-exposed uro-I treated cells lysate, but not in vehicle or uro-I treated cells lysate processed in the dark. Furthermore, data revealed that BiP only aggregated as an artifact of light exposure, not in living cells. Lamin A/C and BiP monomers were not detected in the mass spectral analysis because the gel blocks cut did not include the region where lamin A/C and BiP monomers migrate. Lastly, collagen type I alpha I chain and fibronectin were less abundant in uro-I treated cells lysate processed in the dark compared to control (data not shown). Interestingly, there was no collagen or fibronectin detected in the light processed cell lysate. This confirms that loss of monomer is a reliable read out for protein aggregation and that bone matrix proteins are likely forming high molecular weight aggregates that are unable to migrate into the gel.


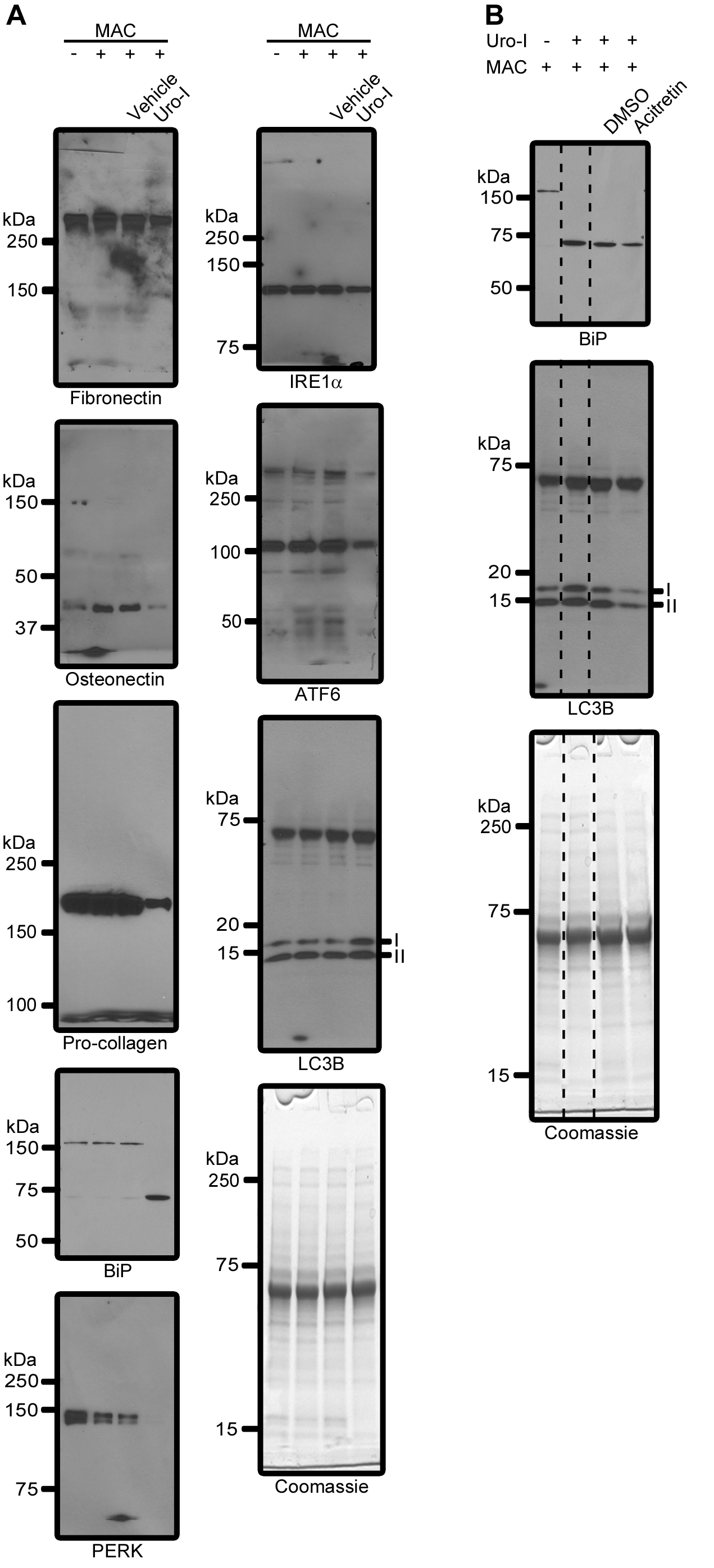


Figure S4. Full-length blots/gels. Uncropped blots and gels from Fig.3C (A) and Fig.3G (B). Membrane/gel edges are shown. Dashed lines represent non-adjacent lanes in the gel.

**SUPPLEMENTARY REFERENCES**

1 Ajioka, R. S., Phillips, J. D. and Kushner, J. P. (2006). Biosynthesis of heme in mammals. Biochimica et Biophysica Acta (BBA) - Molecular Cell Research 1763, 723-736.

2 Layer, G., Reichelt, J., Jahn, D. and Heinz, D. W. (2010). Structure and function of enzymes in heme biosynthesis. Protein Sci 19, 1137-61.

3 Phillips, J. D., Whitby, F. G., Kushner, J. P. and Hill, C. P. (2003). Structural basis for tetrapyrrole coordination by uroporphyrinogen decarboxylase. EMBO J 22, 6225-33.

4 Badminton, M. N. and Elder, G. H. (2014). CHAPTER 28 - The porphyrias: inherited disorders of haem synthesis. In Clinical Biochemistry: Metabolic and Clinical Aspects (Third Edition), (eds W. J. Marshall M. Lapsley A. P. Day and R. M. Ayling), pp. 533-549: Churchill Livingstone.
